# The Present Position of Radiotherapy in Therapeutics
1Read at a meeting of the Society held on January 13th, 1904.


**Published:** 1904-03

**Authors:** W. Kenneth Wills

**Affiliations:** Radiographer and Assistant in the Light Department, Bristol General Hospital


### THE PRESENT POSITION OF RADIOTHERAPY IN THERAPEUTICS.[1]

BY

W. KENNETH WILLS, M.B., B.C. Cantab.,

*Radiographer and Assistant in the Light Department, Bristol General Hospital.*

---

Now that there is a general concession that good work is being done by some or all of the different forms of radiant energy, it may be as well to enquire whether we may be able to detect any theory upon which we may work, and thus remove, if possible, these novel forms of treatment from the limits of absolute empiricism.

[1] Read at a meeting of the Society held on January 13th, 1904.

Whether we use sunlight, the electric arc, the X-ray tube, radium, or the high-frequency apparatus, we find the energy produced differs mainly in the penetrability and quantity of the ultra-violet light or rays of lesser wave-length. The effects produced by any of these methods are similar in kind, though of course much more vigorous with some forms of apparatus than with others.

If we examine a "reaction" we see that at first an erythema occurs, which may pass off without further development; it may, on the other hand, continue to develop into a blister, or the epithelium be only separated on the subsidence of the erythema. With larger dose the epithelial scales may become sodden and come away wholesale, leaving behind little bleeding granulation-like points which tell us of our proximity to the true derma. This might be termed a necrosis of the epithelium.

At first undoubtedly in all forms of this treatment the tissues are exhilarated; later they become depressed. But undoubtedly the healthy cells are more readily invigorated and less easily depressed than diseased cells. Accordingly we see the diseased cells affected adversely first, and it is a frequent phenomenon that after a dose of the X-rays in a case of lupus a marked erythema affects the lupus tissue, while the surrounding healthy tissue is not affected at all.

This can only be explained by a local change, whether vascular or primarily nervous. To my mind it could not be the latter, since to my knowledge no case is on record in which a reaction has spread far beyond the limits of the part exposed and arranged itself according to a nerve distribution. The local stasis, however, indicates an effect upon the vessel walls themselves, or possibly on the nerve end-plates in the vessel walls themselves, which temporarily inhibits them.

We are then in possession of the knowledge that healthy cells are encouraged, and diseased, *i.e.* less highly-organised cells, are depressed.

Now, turning to the therapeutic possibilities of this, we would naturally expect that—

1. Those diseases depending upon a malnutrition of the skin would be benefited by anything which promoted the healthy growth of the healthy cells. In this way we may explain the good wrought in trophic, varicose, and other ulcerations.

2. Again, in cases where ordinarily the skin can, but in the circumstances present cannot deal with disease. In such diseases as in chronic and resistant eczema, psoriasis, and acne we have conditions where it would not be wrong to say that the tendency was always to recovery. But in inveterate cases ray treatment has been found to be of value.

In these two classes the dosage aimed at is such as shall only have stimulating effects.

In a third class I would place those diseases in which partial repair is the rule, but where it is not common to see complete recovery without some outside intervention. Lupus vulgaris, rodent ulcer, and perhaps scirrhus will come under this heading. If then the dosage can be so regulated that the cells of the diseased area can be depressed, while the healthy surrounding cells can be encouraged, the result may be that the diseased cells may be cast off as a slough, as may happen in rodent ulcer, or such a limiting line of demarcation be formed that further spread may be inhibited. The diseased area at the same time is flooded with blood which is of use in lupus, and the cells stimulated to dispose of germs if these are not actually destroyed by the rays.

In this case the rays are required as a selective caustic, and the dosage required has to be measured with the very greatest care.

There is a small class which may well be mentioned, for they have to do with the removal of the hair. Eczema barbæ and sycosis are readily cured under the X-rays, and tinea tonsurans and favus capitis have been reported as more easily treated after the depilation caused by the rays.

In carcinoma it is essentially the "caustic" action which is sought for, an action at the same time which will not destroy the healthy cells.

This brings us to the consideration of the possible treatment of cancer by means of the rays. Undoubtedly the X-rays have a special affinity for epithelial structures. What this affinity consists in is as yet undecided; but undoubtedly effects occur which are not shared by the mesoblastic layers to the same degree. That this alteration produced is progressive and secondarily retrogressive would appear to be the normal. Yet whether the retrogression is procured rapidly enough to arrest or keep pace with the disease is doubtful in many reported cases. It is certainly disappointing to hear of cases which to all intents and purposes are seemingly ideal for the X-ray treatment, and which, though treated carefully and skilfully, show no good result, but occasionally leave a doubt behind as to whether or not the rays have done more harm than good. But yet it remains that some numbers of cases of malignant disease have been reported "cured." It is certainly a great drawback that so many cases of rodent ulcer have been mixed up unclassified in the statistics; but even apart from these a large number seem to be undoubted cases of carcinoma, as definitely stated by the writers. M. Perthes[1] states that thirty-five authors have reported cures of superficial cancers. He himself has had good results with cancer of the breast, and believes that the action does not stop short of the epithelial layers of the skin, but reaches the deeper-seated tumours. Warts, he says, have disappeared, although treated through a sheet of human skin held over them. He believes that there is a preference for epithelial cells, whether normal or cancerous. But it would seem that cancerous epithelium is more easily affected than the normal; for in the experiment of Beckett, quoted by Norman Walker and Gardiner,[2] where he treated simultaneously a small epithelioma, a wart, and an area of healthy skin, the epithelioma started to break down after three exposures and became inflamed; the wart resisted longer, but finally atrophied, and showed reaction in a week; while the normal skin did not react for about a fortnight. This selective action of the rays should certainly be made use of, provided that we can in any way measure the dosage accurately enough to produce the required reaction when desired. This is clearly not work which can be left to the working mechanic or the

[1] *Semaine méd.*, June 10th, 1903.

[2] *Scottish M. and S. J.*, 1903, xii. 265.

electrician, and the employment of such by the profession should be entirely discouraged.

Speaking above of the reported "cures," I added inverted commas. That an ulcerous process will cicatrise over, that a tumour which has been growing rapidly and is painful retrogresses and loses its pain and tenderness, becoming smaller and softer, perhaps becoming no longer distinguishable, would seem to justify the term, ordinarily speaking; but so often in cancer has the most radical removal been disappointing in its ultimate results, that until some "five-year limit" has passed I should personally prefer to speak only of "retrogression." That this retrogression will occur in some cancers treated by the rays is reported upon all hands. I have personally seen nodules retrogress and the general health of the patient improve considerably. In some reported cases flesh has been gained, appetite returned, and the powers of the body restored in a wonderful way; but in others the most patient, careful, and skilled treatment has not had the slightest effect upon the condition. What this variation means is a question for the radio-therapeutists to solve. It may be a variation in treatment, an indiosyncrasy in the patient, the degree of malignancy, or the extent of diffusion of the disease.

In one's own limited experience it is easy to recognise that patients differ very markedly in their reaction to the X-rays. One of my own cases is of interest in this connection. It was a case of lupus vulgaris involving the right side of the neck and face, and for which an operation had been performed previously, resulting in paresis of the seventh nerve. She also had a patch of lupoid ulceration of the hand. I treated these two parts together, arranging them so that as nearly as possible they were equi-distant from the tube. Although one would naturally expect the skin of the face to be more easily affected than that of the hands, the reverse proved to be the case. Indeed, to produce a reaction on the face I had to give much longer exposures than I have had to do in other patients. Whether this muscular paresis had any secondary action on the skin, and hence in some way obstructed the vasomotor reaction, is difficult to say, but the case serves as an instance of how patients may not only differ from one another, but two parts of the same body may react differently. Some patients do not seem to get beyond the first stage of stimulation, and the tumour takes on rapid growth: even, apparently, more or less normal epithelium may be stimulated by the rays and become epitheliomatous. That growth may not only be procured, but also that nutrition may be maintained, is a point alluded to by Codman,[1] who, after raying a case of massive malignant tumour of the breast, could find no trace of degeneration in the centre as is usual. He attributed it to the nutritional effect of the rays. But simultaneously the activity of the surrounding tissue cells is promoted, and the appearance of shrunken cancer cells in the granulation tissue seems to be the outcome of the healthy tissues invading the cancerous. If, however, the dose could be so accurately increased as to depress the malignant cells while the healthy cells were still only excited, then should we have a means at our disposal of successfully treating cancer.

If this is successfully carried out a retrogression follows, and unless an error is made in dosage subsequently a "cure" becomes possible. At the present date, however, our methods of determining accuracy of dosage are so inadequate that, if I may be allowed the expression, a retrogression is a fortunate accident.

In rodent ulcer the balance is not quite so delicately swung, owing to the lower malignancy of the tissues. But even here we meet fulminating cases which suddenly become abnormally active, and the constitutional disturbances are severe. The patient becomes sallow in complexion, with weak, rapid pulse, appetite poor, and feels intensely weak. "*Cachexia*" has become acute, the cancerous products being absorbed with consequent toxæmia.

The evidence of the microscope does not disprove this theory of the action of the rays, though it is somewhat conflicting. For while Shattock[2] has examined a tumour which had been under the rays for three months, and has found no change attributable to the rays either macro- or microscopically,

[1] *Hospital Mag.*, May, 1903.

[2] *Lancet*, 1902, ii. 1,541.

other observers[1] have observed irregular behaviour of cells, indicating degeneration in some form or another according to the degree of reaction obtained.

Little has been done up to the present in primary and operable cases of cancer, and there seems to be as yet little excuse for so doing, judging by results. But yet some cases have been recorded, and Morton,[2] who has had a large experience in X-ray work, is now recommending the treatment of cases before operation as well as after. He does not believe that "valuable time will be lost," and provided that the X-ray treatment be only adopted so long as the patient makes decided progress towards recovery, he holds that it may be used in primary cases without operation. He has seen in breast cases infected, indurated, swollen and sensitive lymphatics and glands become to all intents and purposes normal. "What," he asks, "better preparation can there be for operative procedure?" He even goes further than this, and states that "cancerous disease in its incipiency, when not too deeply placed, is decisively arrested in its growth and caused to disappear." Although such a hopeful view of the case has not been substantiated by any cases I have met with personally, it is only fit in a paper like the present to mention the confidence felt in the treatment in some quarters.

Dr. Bashford,[3] of the Cancer "Research Fund," gives his opinion as follows:—"There is a general consensus of opinion that many superficial rodent ulcers, even if very extensive and of long standing, can be healed completely by X-rays alone. Recurrences occur in 20 to 40 per cent. of cases, but these are amenable to treatment as the primary conditions were."

Complete healing is reported in 141 out of 216 cases = 65 per cent. successes; improvements in 43; no benefit in 16; disease aggravated in 3 cases.

As far as malignant growths other than rodent ulcer are concerned, he says:—"The results so far brought to our notice do not establish the efficiency of any of these measures (to wit, the

[1] Ellis, *Am. J. M. Sc.*, January, 1903; Scholtz, *Brit. J. Dermat.*, 1902, xiv. 397; Carl Beck, *Ibid.*, p. 399; Stewart, *Med. Rec.*, 1903, lxiii. 837, and others.

[2] *Med. Rec.*, 1903, lxiii. 845. [3] *Brit. M. J.*, August 8th, 1903.

Finsen light, high-frequency electric currents, and X-ray) as curative agents in sarcoma and carcinoma. The whole question is *sub judice.*"

However, if we are not to look forward actually with confidence towards recovery in cases of cancer, we may see any of the following results which Morton has given in a summary:—

1. Relief from excruciating pain and constant suffering—often immediate.
2. Reduction in size of new growths.
3. Establishment of process of repair.
4. Removal of odour if present.
5. Cessation of discharge.
6. Softening and disappearance of lymphatic nodes.
7. Removal of cachectic colour and appearance of skin.
8. Improvement of general health.

And as such results may occur, the rays must be acknowledged a place as a palliative, and as such a valuable addition to our armamentarium.

